# Role of High Flow Nasal Cannula as a Novel Therapy for Treatment of Severe Obstructive Sleep Apnea in a Child With Neuroendocrine Hyperplasia of Infancy: A Case Report and Review of Literature

**DOI:** 10.7759/cureus.60091

**Published:** 2024-05-11

**Authors:** Claire Feller, Scott Bickel, Rajaneeshankar Palani, Egambaram Senthilvel

**Affiliations:** 1 Pediatrics, University of Louisville School of Medicine, Louisville, USA; 2 Pediatric Radiology, Norton Children’s Hospital, Louisville, USA; 3 Pediatrics, University of Louisville, Louisville, USA

**Keywords:** obstructive sleep apnea, neuroendocrine, infancy, hyperplasia, high flow nasal canula

## Abstract

Neuroendocrine hyperplasia of infancy is a rare form of pediatric interstitial lung disease presenting with hypoxemia, tachypnea, retractions, and persistent pulmonary crackles in the first year of life. As these children frequently require supplemental oxygen therapy and demonstrate nighttime hypoxemia, there is a concern for an increased prevalence of sleep-disordered breathing in this population, including obstructive sleep apnea. As untreated sleep-disordered breathing is associated with adverse developmental outcomes for children, it is essential to promptly diagnose and treat. However, treatment of obstructive sleep apnea is often challenging in children. In this report, we describe a case of a child diagnosed with neuroendocrine hyperplasia of infancy at 12 months of age who was subsequently found to have severe obstructive sleep apnea that persisted despite adenotonsillectomy. As continuous positive airway pressure was not well tolerated, the patient was initiated on a high-flow nasal cannula at nighttime, which resulted in improvement of his sleep apnea and daytime functioning with better adherence to treatment. Our case illustrates the importance of screening for sleep-disordered breathing in patients with neuroendocrine hyperplasia of infancy, as well as the utility of a high-flow nasal cannula as a novel, effective treatment for pediatric obstructive sleep apnea.

## Introduction

Neuroendocrine hyperplasia of infancy (NEHI) is a rare form of pediatric interstitial lung disease first described in 2005 that is associated with the clinical syndrome known as persistent tachypnea of infancy [1]. Symptoms begin in early infancy with a mean age of onset of three months and present with persistent tachypnea, episodes of hypoxemia, retractions, and crackles on exam [1-3]. Children with NEHI may exhibit sleep-disordered breathing including obstructive sleep apnea (OSA), thus necessitating polysomnogram (PSG) evaluation [4]. While the first-line treatment of pediatric OSA is adenotonsillectomy, persistent OSA may require continuous positive airway pressure (CPAP) therapy [5]. While beneficial in treating OSA, CPAP therapy can pose significant challenges in adherence for children. Recent studies have demonstrated the role of high-flow nasal cannula (HFNC) in the treatment of OSA in infants and children [6-9]. In this case report, we discuss the evaluation and treatment of severe OSA in a child with NEHI and review the role of HFNC in the treatment of pediatric OSA.

## Case presentation

A male infant was diagnosed with NEHI at the age of 12 months. He initially presented in infancy with a history of coughing, congestion, shortness of breath with tachypnea, and failure to thrive. Diagnosis of NEHI was subsequently confirmed by lung biopsy. At 13 months of life, he was noted to have retractions while sleeping despite receiving home supplemental oxygen, raising concern for sleep-disordered breathing. Thus, he was referred to pediatric otorhinolaryngology for flexible and rigid bronchoscopy, which noted ankyloglossia with a normal-appearing upper airway. At age 2, he underwent his first PSG (Table 1) and was found to have severe OSA with an apnea-hypopnea index (AHI) of 20 and rapid eye movement (REM) AHI of 81. Oxygen desaturation nadir was 38% on room air, and 394.5 minutes were spent with oxygen saturation below 89%. Carbon dioxide was not elevated.

**Table 1:** Polysomnographic findings at different ages PSG = polysomnogram; T&A = tonsillectomy and adenoidectomy; REM = rapid eye movement

PSG Variables	Baseline PSG	Post T&A PSG	PSG at age 4	PSG at age 8	PSG at age 11
Sleep efficiency, %	79.6	87.7	98.5	92.7	99.0
Sleep onset latency, min	1.0	0.4	3.1	2	5.0
Wake after sleep onset	107.7	55.5	4.8	35.7	0.0
REM onset latency, min	113	42.5	65.0	141.5	121.0
Stage N1 sleep, %	0.8	0.4	0.0	3.5	4.0
Stage N2 sleep, %	65.9	51.7	45.7	42.5	38.3
Stage N3 sleep, %	24.2	23.7	29.4	31.6	30.9
Stage REM sleep, %	9.1	24.3	24.9	22.3	26.8
Obstructive apneas	0	4	1	5	1
Obstructive hypopneas	141	93	95	74	82
Central apneas	0	2	1	0	0
Apnea-hypopnea index/hour	20.0	14.9	11.1	10.0	9.8
REM apnea-hypopnea index/hour	81.0	53.2	43.2	40.9	32.8
Obstructive apnea-hypopnea index	20.0	14.6	11.1	10.0	9.8
Central apnea index/h	0	0.3	0.1	0	0
Arousal index, events/h	23.5	8.1	14.7	9.8	6.3
Oxygen saturation nadir, %	38	78	78	80	84
Time spent in oxygen saturation below 89%	394.5	26.4	27.2	20.0	4.0
Carbon dioxide maximum, mm Hg	49	51	60	64	51

**Figure 1:**
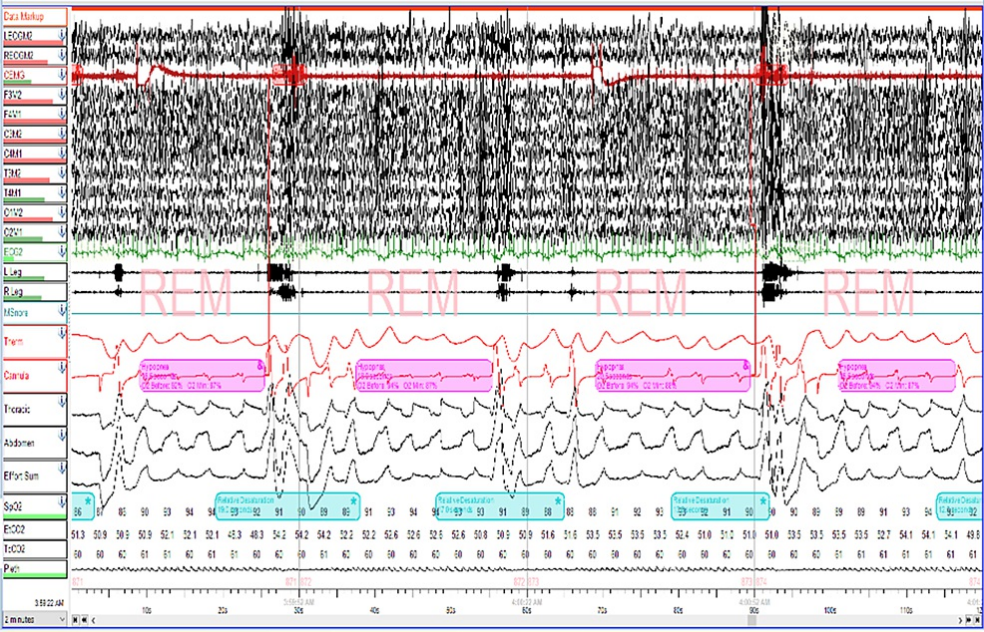
Two minutes epoch of baseline polysomnogram showing hypopnea during REM sleep REM = Rapid eye movement

He underwent adenotonsillectomy at that time. However, post-operative follow-up PSG (Table 1) showed significant residual OSA in REM sleep with AHI of 14.9, REM AHI of 53.2, and most of the events were obstructive hypopneas (Figure 1). However, oxygen desaturation significantly improved. Oxygen desaturation nadir was 78%, and 26.4 minutes were spent in oxygen desaturation below 89%. While CPAP was initially attempted, it was not tolerated by the patient. Therefore, parents decided to continue to use 2 L/min of supplemental oxygen via nasal cannula at night to correct his nighttime hypoxemia. Echocardiography was performed annually due to NEHI and did not demonstrate any concern for pulmonary hypertension.

**Figure 2:**
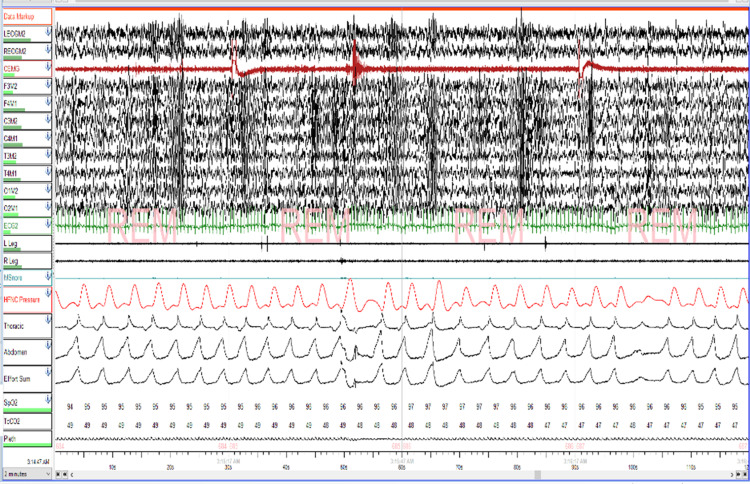
High-flow nasal cannula (HFNC) titration polysomnogram, 2 minutes epoch showing normalization of respiratory events during REM sleep REM = rapid eye movement

**Figure 3:**
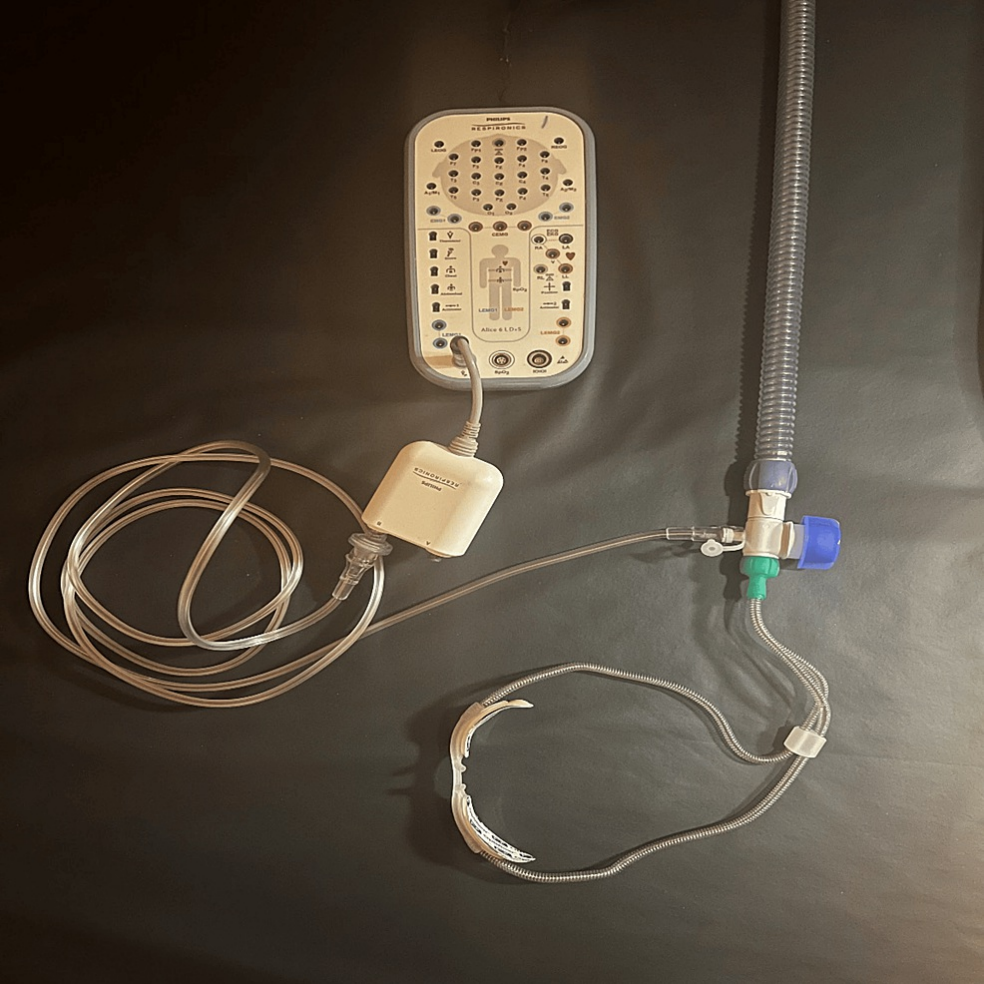
High-flow nasal cannula (HFNC) sleep study integration The HFNC equipment configuration used to acquire pressure signal for a high-flow nasal cannula titration is pictured above. Signal was achieved by utilizing the Fisher & Paykel OptiFlow Junior adapter within the OptiFlow breathing circuit through the provided port for supplemental oxygen entrainment. Extension tubing was connected at this port and attached to the pressure transducer module in the Philips Respironics Alice 6 headbox. The pressure transducer channel label within Sleepware G3 software recording was changed to “HFNC Pressure” for channel identification. Pressure changes were made manually within the clinical settings of the HFNC device and documented in the study during the titration. For signal analysis, a low pass filter setting of 0.4Hz was applied to the created channel.

At six years of life, his severe OSA remained treated with oxygen alone, and he started to have hypoventilation as well, which prompted us to reconsider positive airway pressure therapy. Since he did not tolerate the CPAP in the past, the patient was trialed on HFNC via the Airvo-2 humidifier with an integrated flow generator (Fisher & Paykel, Auckland, New Zealand). An HFNC titration study was performed (Figures 2, 3), which showed that at 15 liters/min (LPM) with 1 LPM of supplemental oxygen, the patient’s AHI improved to 5.8 (14.6 in REM) with a mean SpO2 of 97%, with dramatic improvement in the patient’s baseline hypoxemia as well. Parents reported that his sleep, daytime functioning, and school performance were improved significantly after the initiation of HFNC.

**Figure 4:**
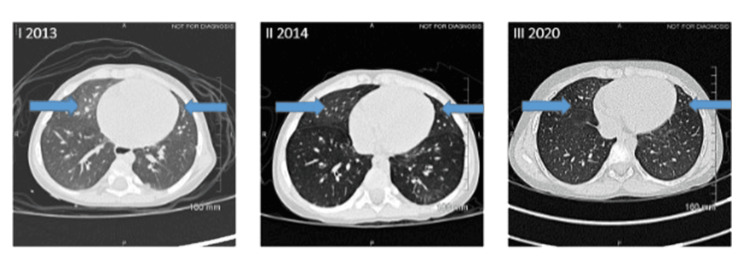
High resolution non-contrast computed tomography of the chest: Thin axial sections show the persistence of ground glass densities in the right middle lobe and lingula in 2013, 2014 and 2020.

Thus, the patient tolerated home HFNC well with improvement in OSA from baseline. At age 8, follow-up PSG (Table 1) demonstrated an overall AHI of 10.0 and REM AHI of 40.9, with oxygen desaturation nadir of 80%. Most recent PSG (Table 1) demonstrated moderate OSA (overall AHI of 9.8) with most events occurring during REM sleep (REM AHI of 32.8). Oxygen nadir was noted to be 84% on room air, with a mean SaO2 of 95%. He currently remains stable on 35 LPM after having another HFNC titration study and is now on room air. He was also seen by an otolaryngologist for the persistence of OSA after adenotonsillectomy and had a drug-induced sleep endoscopy at age 8 years, which showed some anteroposterior collapse at the oropharynx and epiglottic level. He had follow-up high-resolution computed tomography, which showed persistent ground glass densities in the right middle lobe and lingula in 2013, 2014 and 2020 (Figure 4), consistent with typical NEHI findings.

## Discussion

NEHI was first described by Deterding et al. in 2005, who identified a group of children presenting with clinical symptoms of interstitial lung disease and were subsequently found to have hyperplasia of pulmonary neuroendocrine cell (PNEC) on lung biopsy [1]. Diagnosis is supported by the presence of persistent pulmonary symptoms in infancy including retractions, crackles, and hypoxia, as well as a high-resolution computed tomography (CT) scan demonstrating ground-glass opacification and air trapping [1,10]. While the majority of children require supplemental oxygen at diagnosis, these children generally experience disease improvement with time and often can be eventually weaned off oxygen [1, 3, 11].

The role of polysomnography in this population has been increasingly recognized. In their retrospective study including 77 patients with NEHI, Liptzin et al. reported that of the 14 patients who underwent PSG, 57% were found to have OSA [4]. While many children with NEHI exhibit hypoxemia during sleep, they may also demonstrate other sleep-related pathologies including central sleep apnea and periodic limb movement disorder, as well as poor sleep efficiency [4]. If left untreated, pediatric sleep-disordered breathing (SDB) is associated with long-term adverse cognitive, cardiovascular, and metabolic effects [12-15]. Thus, PSG may be a useful diagnostic tool in children with NEHI.

As demonstrated by the present case, HFNC has been increasingly recognized as a potential treatment for OSA in children. In their retrospective review of 22 children with OSA treated with HFNC, Ignatiuk et al. found that children treated with HFNC demonstrated significant improvement in obstructive apnea-hypopnea indices (OAHI) and that the majority of their patients tolerated HFNC well [6]. They also noted an improvement in both oxygen and carbon dioxide levels after treatment with HFNC and proposed that HFNC may also have a role in reducing hypoventilation during sleep [6]. Kwok et al. noted a significant improvement in oxygenation as well as a decrease in the number of respiratory events in 10 out of 15 infants with OSA who were treated with heated humidified HFNC and proposed it as an effective alternative to nasal CPAP [7]. Similarly, in their systematic review and meta-analysis that included a total of 67 children, Du et al. noted a significant improvement in OAHI as well as obstructive hypopnea index (OHI) and obstructive apnea index (OAI) after treatment with HFNC, remarking that despite the small sample sizes of the individual studies included, these results remained consistent [8].

When compared with CPAP, HFNC demonstrates similar efficacy. A randomized cross-over trial performed by Fishman et al. examined 18 children diagnosed with OSA, each of which completed both an HFNC titration study as well as a CPAP titration study in a randomized order [9]. They noted no significant difference in OAHI reduction from baseline with HFNC versus CPAP and suggest that as they have similar clinical benefits, HFNC may be a preferred option for children with poor CPAP adherence [9]. In the case presented above, while the patient was unable to tolerate CPAP for his severe OSA, HFNC provided an effective option with improved adherence, which led to improved control of his OSA.

## Conclusions

This case of a child with NEHI who presented with severe OSA despite adenotonsillectomy and inability to tolerate CPAP demonstrates the utility of HFNC in the treatment of pediatric OSA. As NEHI is increasingly recognized as a pediatric interstitial lung disease, we suggest that physicians maintain a low threshold for PSG evaluation in this population, as sleep-disordered breathing may be more prevalent in these patients compared to their healthy counterparts. Given the potential for adverse neurocognitive, cardiovascular, and metabolic effects in children with untreated sleep-disordered breathing, it is essential to promptly evaluate and treat pediatric patients with concern for OSA. As HFNC demonstrates similar efficacy to CPAP, we recommend that pediatric providers consider HFNC as an alternative treatment for OSA in patients unable to tolerate CPAP.
